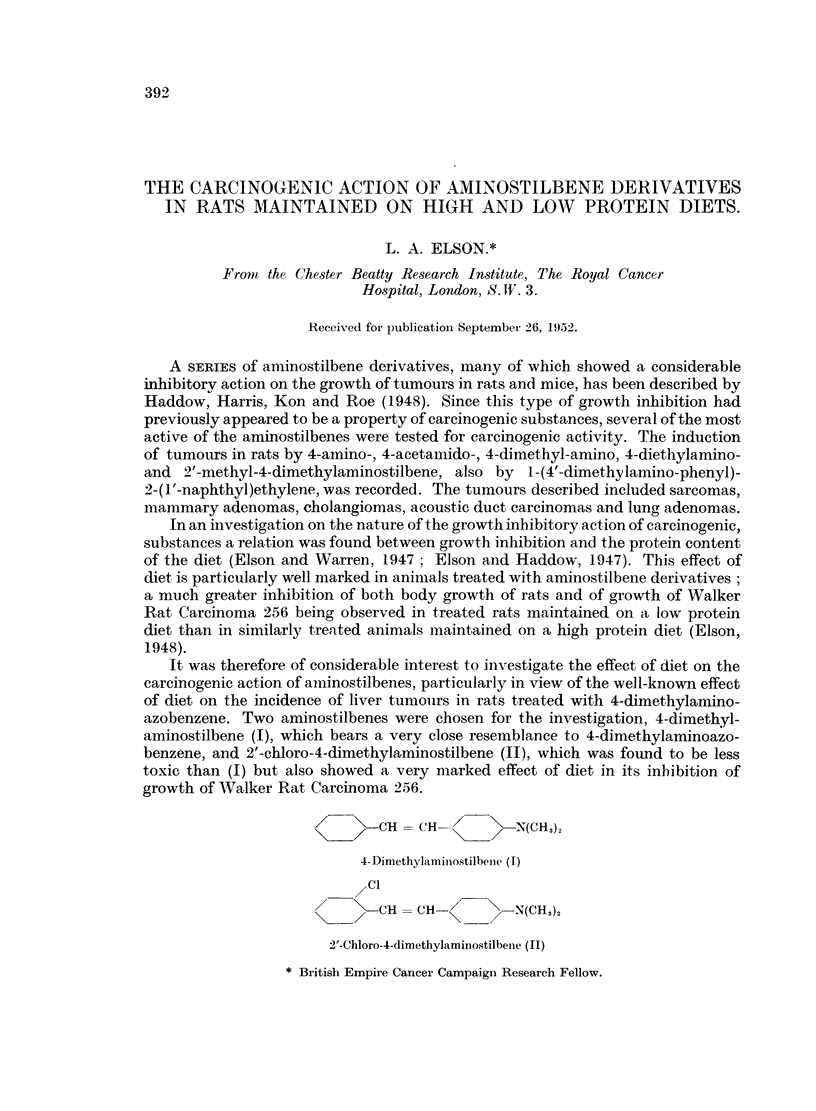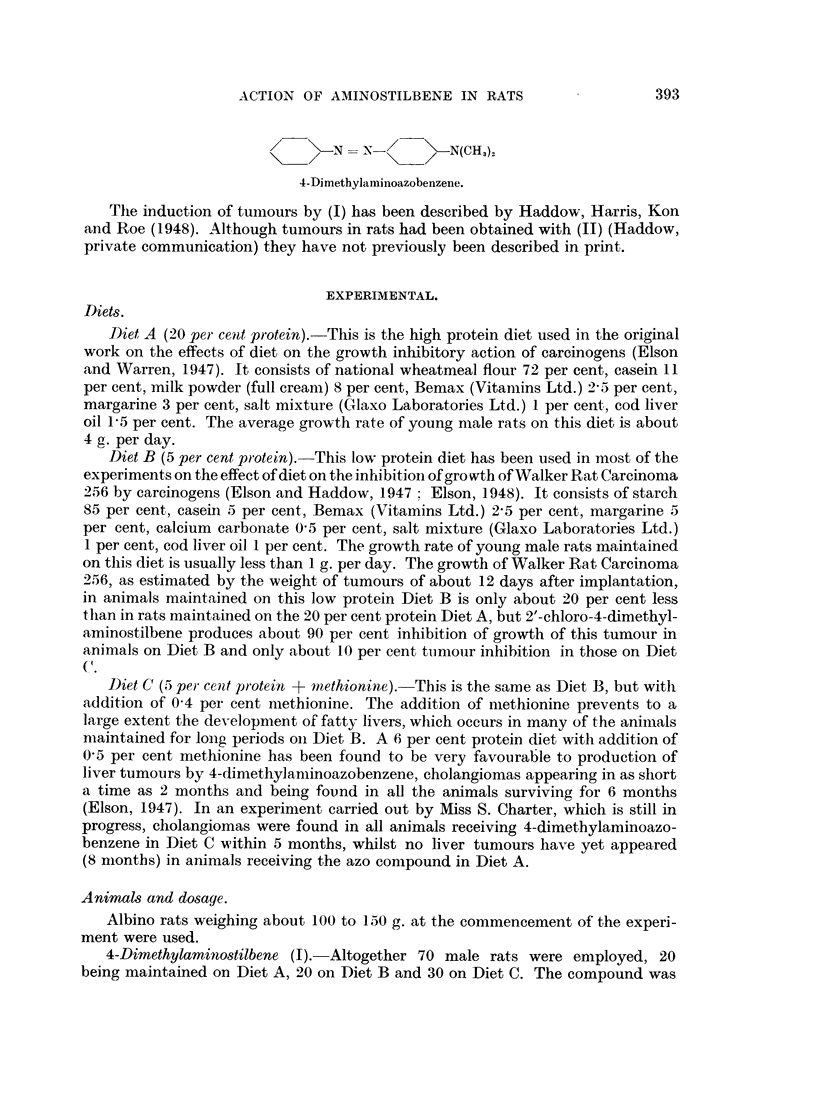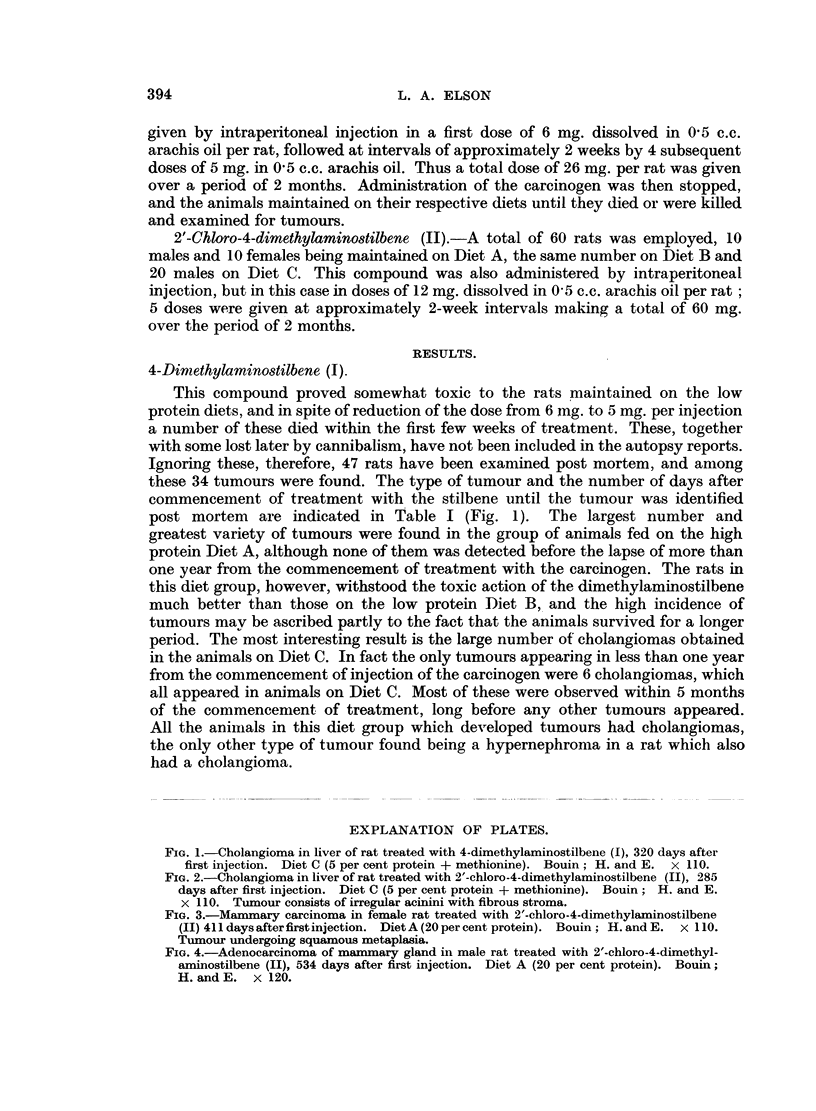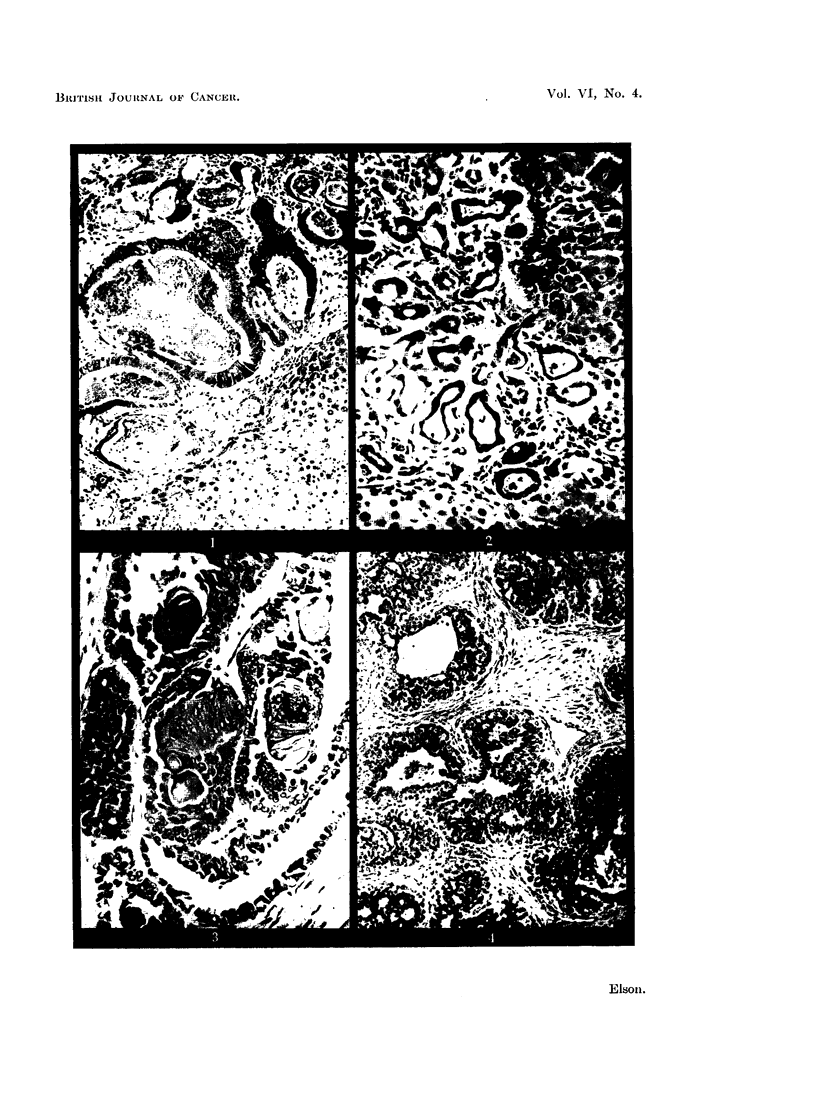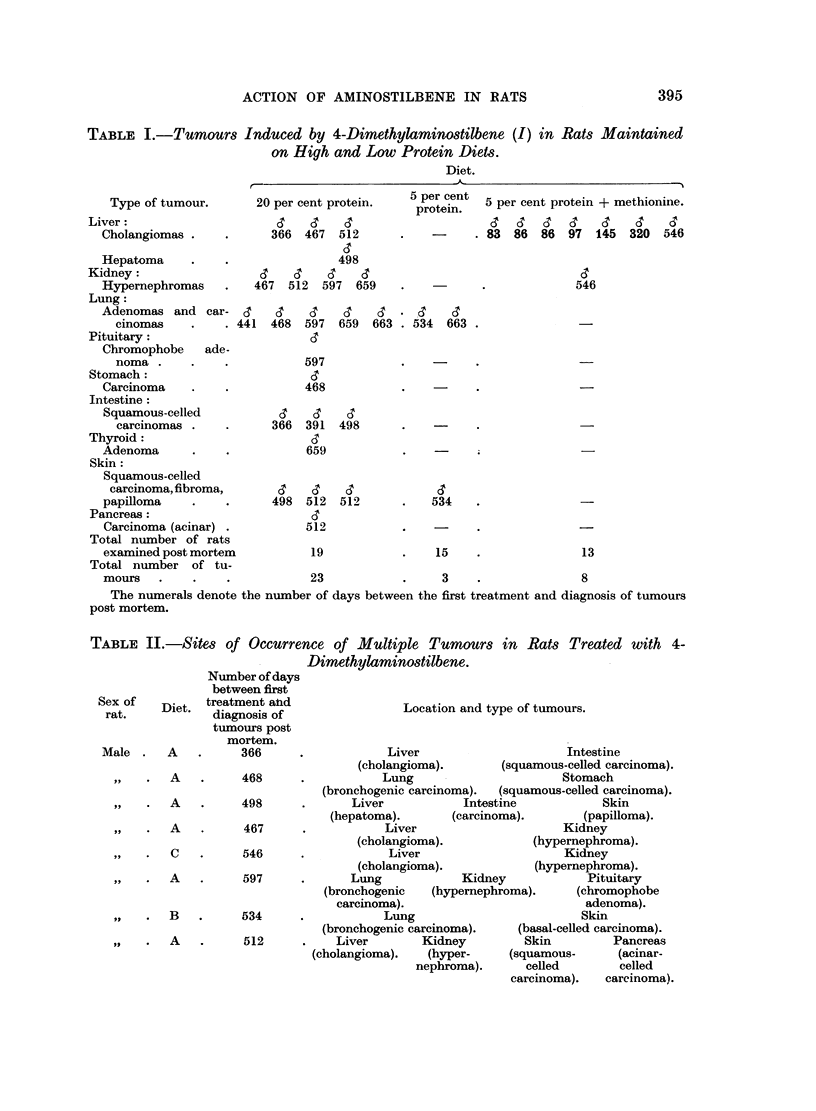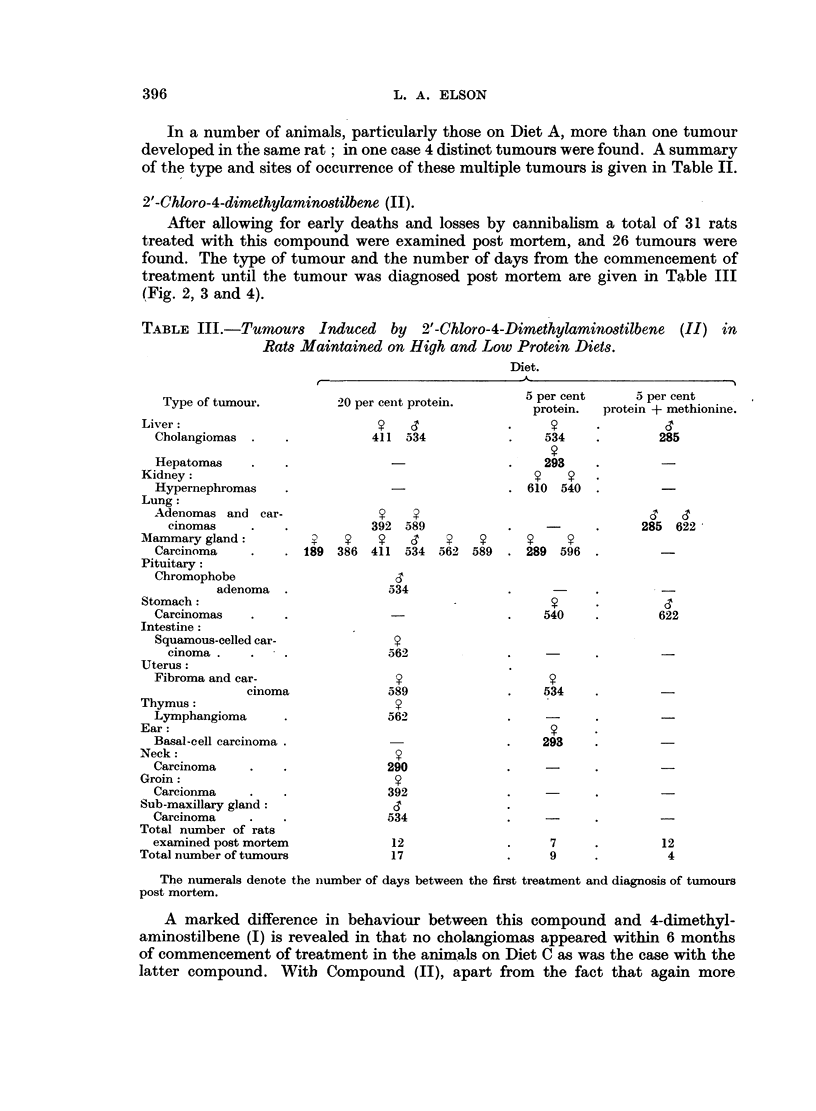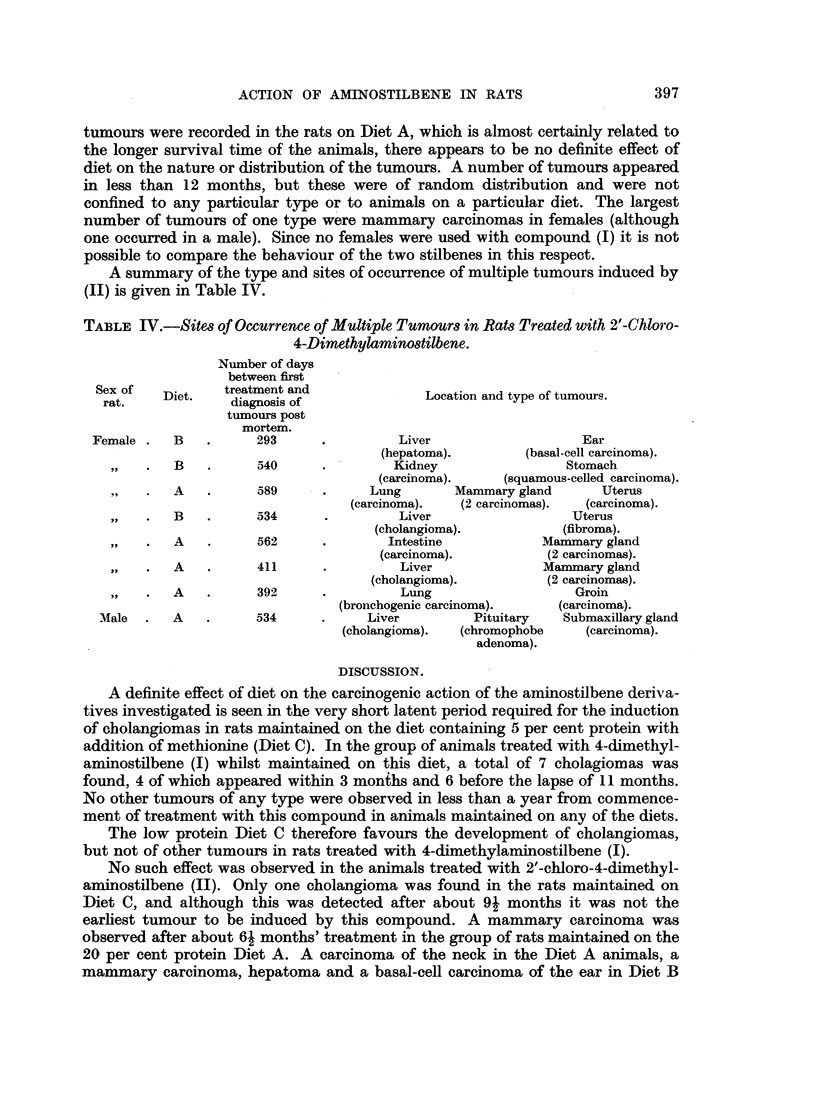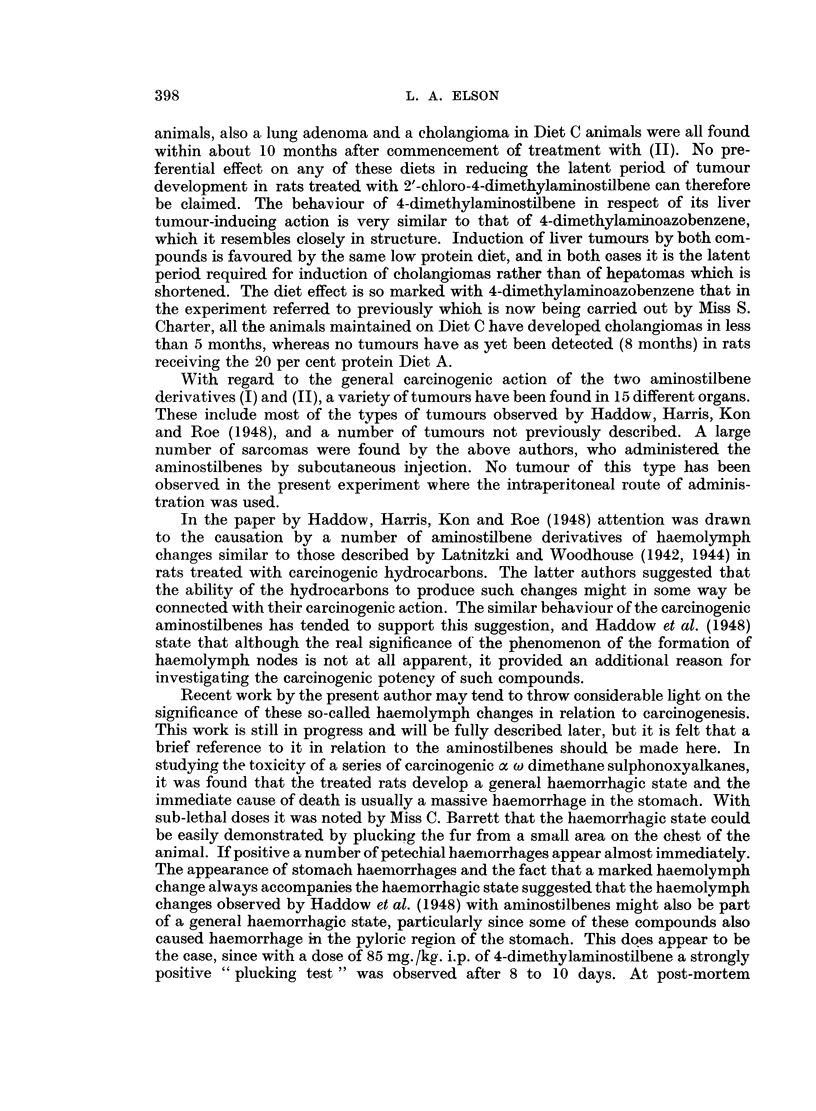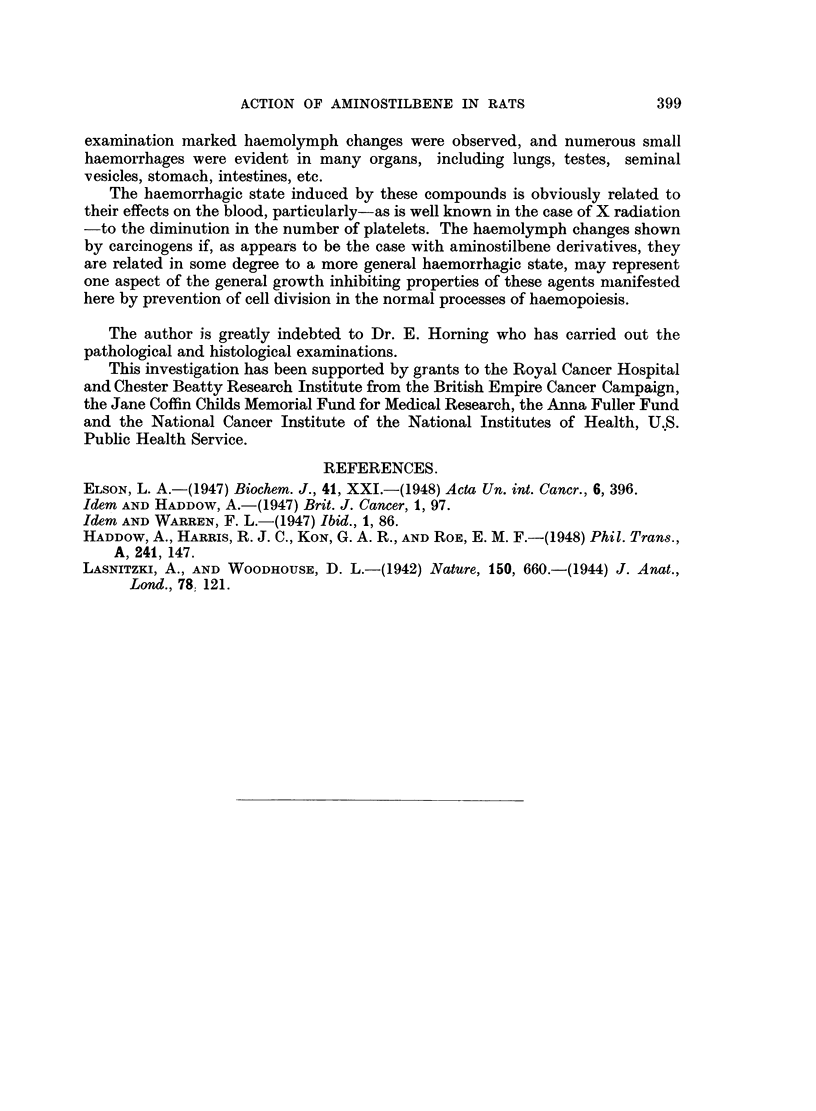# The Carcinogenic Action of Aminostilbene Derivatives in Rats Maintained on High and Low Protein Diets

**DOI:** 10.1038/bjc.1952.44

**Published:** 1952-12

**Authors:** L. A. Elson

## Abstract

**Images:**


### THE CARCINOGENIC ACTION OF AMINOSTILBENE DERIVATIVES IN RATS MAINTAINED ON HIGH AND LOW PROTEIN DIETS.

L. A. ELSON.*

*From the Chester Beatty Research Institute, The Royal Cancer Hospital, London, S.W. 3.*

Received for publication September 26, 1952.

A SERIES of aminostilbene derivatives, many of which showed a considerable inhibitory action on the growth of tumours in rats and mice, has been described by Haddow, Harris, Kon and Roe (1948). Since this type of growth inhibition had previously appeared to be a property of carcinogenic substances, several of the most active of the aminostilbenes were tested for carcinogenic activity. The induction of tumours in rats by 4-amino-, 4-acetamido-, 4-dimethyl-amino, 4-diethylamino- and 2′-methyl-4-dimethylaminostilbene, also by 1-(4′-dimethylamino-phenyl)-2-(1′-naphthyl)ethylene, was recorded. The tumours described included sarcomas, mammary adenomas, cholangiomas, acoustic duct carcinomas and lung adenomas.

In an investigation on the nature of the growth inhibitory action of carcinogenic, substances a relation was found between growth inhibition and the protein content of the diet (Elson and Warren, 1947 ; Elson and Haddow, 1947). This effect of diet is particularly well marked in animals treated with aminostilbene derivatives ; a much greater inhibition of both body growth of rats and of growth of Walker Rat Carcinoma 256 being observed in treated rats maintained on a low protein diet than in similarly treated animals maintained on a high protein diet (Elson, 1948).

It was therefore of considerable interest to investigate the effect of diet on the carcinogenic action of aminostilbenes, particularly in view of the well-known effect of diet on the incidence of liver tumours in rats treated with 4-dimethylamino-azobenzene. Two aminostilbenes were chosen for the investigation, 4-dimethyl-aminostilbene (I), which bears a very close resemblance to 4-dimethylaminoazo-benzene, and 2′-chloro-4-dimethylaminostilbene (II), which was found to be less toxic than (I) but also showed a very marked effect of diet in its inhibition of growth of Walker Rat Carcinoma 256.

4-Dimethylaminostilbene (I)

2′-Chloro-4-dimethylaminostilbene (II)

* British Empire Cancer Campaign Research Fellow.

4-Dimethylaminoazobenzene.

The induction of tumours by (I) has been described by Haddow, Harris, Kon and Roe (1948). Although tumours in rats had been obtained with (II) (Haddow, private communication) they have not previously been described in print.

### EXPERIMENTAL.

### *Diets.*

*Diet A* (20 *per cent protein*).—This is the high protein diet used in the original work on the effects of diet on the growth inhibitory action of carcinogens (Elson and Warren, 1947). It consists of national wheatmeal flour 72 per cent, casein 11 per cent, milk powder (full cream) 8 per cent, Bemax (Vitamins Ltd.) 2·5 per cent, margarine 3 per cent, salt mixture (Glaxo Laboratories Ltd.) 1 per cent, cod liver oil 1·5 per cent. The average growth rate of young male rats on this diet is about 4 g. per day.

*Diet B* (5 *per cent protein*).—This low protein diet has been used in most of the experiments on the effect of diet on the inhibition of growth of Walker Rat Carcinoma 256 by carcinogens (Elson and Haddow, 1947 ; Elson, 1948). It consists of starch 85 per cent, casein 5 per cent, Bemax (Vitamins Ltd.) 2·5 per cent, margarine 5 per cent, calcium carbonate 0·5 per cent, salt mixture (Glaxo Laboratories Ltd.) 1 per cent, cod liver oil 1 per cent. The growth rate of young male rats maintained on this diet is usually less than 1 g. per day. The growth of Walker Rat Carcinoma 256, as estimated by the weight of tumours of about 12 days after implantation, in animals maintained on this low protein Diet B is only about 20 per cent less than in rats maintained on the 20 per cent protein Diet A, but 2′-chloro-4-dimethyl-aminostilbene produces about 90 per cent inhibition of growth of this tumour in animals on Diet B and only about 10 per cent tumour inhibition in those on Diet C.

*Diet C* (5 *per cent protein* + *methionine*).—This is the same as Diet B, but with addition of 0·4 per cent methionine. The addition of methionine prevents to a large extent the development of fatty livers, which occurs in many of the animals maintained for long periods on Diet B. A 6 per cent protein diet with addition of 0·5 per cent methionine has been found to be very favourable to production of liver tumours by 4-dimethylaminoazobenzene, cholangiomas appearing in as short a time as 2 months and being found in all the animals surviving for 6 months (Elson, 1947). In an experiment carried out by Miss S. Charter, which is still in progress, cholangiomas were found in all animals receiving 4-dimethylaminoazo-benzene in Diet C within 5 months, whilst no liver tumours have yet appeared (8 months) in animals receiving the azo compound in Diet A.

### *Animals and dosage.*

Albino rats weighing about 100 to 150 g. at the commencement of the experiment were used.

4-*Dimethylaminostilbene* (I).—Altogether 70 male rats were employed, 20 being maintained on Diet A, 20 on Diet B and 30 on Diet C. The compound was given by intraperitoneal injection in a first dose of 6 mg. dissolved in 0·5 c.c. arachis oil per rat, followed at intervals of approximately 2 weeks by 4 subsequent doses of 5 mg. in 0·5 c.c. arachis oil. Thus a total dose of 26 mg. per rat was given over a period of 2 months. Administration of the carcinogen was then stopped, and the animals maintained on their respective diets until they died or were killed and examined for tumours.

2′-*Chloro*-4-*dimethylaminostilbene* (II).—A total of 60 rats was employed, 10 males and 10 females being maintained on Diet A, the same number on Diet B and 20 males on Diet C. This compound was also administered by intraperitoneal injection, but in this case in doses of 12 mg. dissolved in 0·5 c.c. arachis oil per rat; 5 doses were given at approximately 2-week intervals making a total of 60 mg. over the period of 2 months.

### RESULTS.

4-*Dimethylaminostilbene* (I).

This compound proved somewhat toxic to the rats maintained on the low protein diets, and in spite of reduction of the dose from 6 mg. to 5 mg. per injection a number of these died within the first few weeks of treatment. These, together with some lost later by cannibalism, have not been included in the autopsy reports. Ignoring these, therefore, 47 rats have been examined post mortem, and among these 34 tumours were found. The type of tumour and the number of days after commencement of treatment with the stilbene until the tumour was identified post mortem are indicated in Table I (Fig. 1). The largest number and greatest variety of tumours were found in the group of animals fed on the high protein Diet A, although none of them was detected before the lapse of more than one year from the commencement of treatment with the carcinogen. The rats in this diet group, however, withstood the toxic action of the dimethylaminostilbene much better than those on the low protein Diet B, and the high incidence of tumours may be ascribed partly to the fact that the animals survived for a longer period. The most interesting result is the large number of cholangiomas obtained in the animals on Diet C. In fact the only tumours appearing in less than one year from the commencement of injection of the carcinogen were 6 cholangiomas, which all appeared in animals on Diet C. Most of these were observed within 5 months of the commencement of treatment, long before any other tumours appeared. All the animals in this diet group which developed tumours had cholangiomas, the only other type of tumour found being a hypernephroma in a rat which also had a cholangioma.

---

### EXPLANATION OF PLATES.

FIG. 1.—Cholangioma in liver of rat treated with 4-dimethylaminostilbene (I), 320 days after first injection. Diet C (5 per cent protein + methionine). Bouin; H. and E. × 110.

FIG. 2.—Cholangioma in liver of rat treated with 2′-chloro-4-dimethylaminostilbene (II), 285 days after first injection. Diet C (5 per cent protein + methionine). Bouin; H. and E. × 110. Tumour consists of irregular acinini with fibrous stroma.

FIG. 3.—Mammary carcinoma in female rat treated with 2′-chloro-4-dimethylaminostilbene (II) 411 days after first injection. Diet A (20 per cent protein). Bouin; H. and E. × 110. Tumour undergoing squamous metaplasia.

FIG. 4.—Adenocarcinoma of mammary gland in male rat treated with 2′-chloro-4-dimethylaminostilbene (II), 534 days after first injection. Diet A (20 per cent protein). Bouin; H. and E. × 120.

1

2

3

4

Elson.

TABLE I.—*Tumours Induced by 4-Dimethylaminostilbene (I) in Rats Maintained on High and Low Protein Diets.*

| Type of tumour. | Diet. 20 per cent protein. | 5 per cent protein. | 5 per cent protein + methionine. |
|---|---|---|---|
| Liver : | | | |
| Cholangiomas | ♂ 366, ♂ 467, ♂ 512 | — | ♂ **83**, ♂ **86**, ♂ **86**, ♂ **97**, ♂ **145**, ♂ **320**, ♂ 546 |
| Hepatoma | ♂ 498 | | |
| Kidney : | | | |
| Hypernephromas | ♂ 467, ♂ 512, ♂ 597, ♂ 659 | — | ♂ 546 |
| Lung : | | | |
| Adenomas and carcinomas | ♂ 441, ♂ 468, ♂ 597, ♂ 659, ♂ 663 | ♂ 534, ♂ 663 | — |
| Pituitary : | | | |
| Chromophobe adenoma | ♂ 597 | — | — |
| Stomach : | | | |
| Carcinoma | ♂ 468 | — | — |
| Intestine : | | | |
| Squamous-celled carcinomas | ♂ 366, ♂ 391, ♂ 498 | — | — |
| Thyroid : | | | |
| Adenoma | ♂ 659 | — | — |
| Skin : | | | |
| Squamous-celled carcinoma, fibroma, papilloma | ♂ 498, ♂ 512, ♂ 512 | ♂ 534 | — |
| Pancreas : | | | |
| Carcinoma (acinar) | ♂ 512 | — | — |
| Total number of rats examined post mortem | 19 | 15 | 13 |
| Total number of tumours | 23 | 3 | 8 |

The numerals denote the number of days between the first treatment and diagnosis of tumours post mortem.

TABLE II.—*Sites of Occurrence of Multiple Tumours in Rats Treated with 4-Dimethylaminostilbene.*

| Sex of rat. | Diet. | Number of days between first treatment and diagnosis of tumours post mortem. | Location and type of tumours. |
|---|---|---|---|
| Male | A | 366 | Liver (cholangioma). Intestine (squamous-celled carcinoma). |
| ,, | A | 468 | Lung (bronchogenic carcinoma). Stomach (squamous-celled carcinoma). |
| ,, | A | 498 | Liver (hepatoma). Intestine (carcinoma). Skin (papilloma). |
| ,, | A | 467 | Liver (cholangioma). Kidney (hypernephroma). |
| ,, | C | 546 | Liver (cholangioma). Kidney (hypernephroma). |
| ,, | A | 597 | Lung (bronchogenic carcinoma). Kidney (hypernephroma). Pituitary (chromophobe adenoma). |
| ,, | B | 534 | Lung (bronchogenic carcinoma). Skin (basal-celled carcinoma). |
| ,, | A | 512 | Liver (cholangioma). Kidney (hypernephroma). Skin (squamous-celled carcinoma). Pancreas (acinar-celled carcinoma). |

In a number of animals, particularly those on Diet A, more than one tumour developed in the same rat ; in one case 4 distinct tumours were found. A summary of the type and sites of occurrence of these multiple tumours is given in Table II.

*2′-Chloro-4-dimethylaminostilbene* (II).

After allowing for early deaths and losses by cannibalism a total of 31 rats treated with this compound were examined post mortem, and 26 tumours were found. The type of tumour and the number of days from the commencement of treatment until the tumour was diagnosed post mortem are given in Table III (Fig. 2, 3 and 4).

TABLE III.—*Tumours Induced by 2′-Chloro-4-Dimethylaminostilbene (II) in Rats Maintained on High and Low Protein Diets.*

| Type of tumour. | Diet. 20 per cent protein. | 5 per cent protein. | 5 per cent protein + methionine. |
|---|---|---|---|
| Liver : | | | |
| Cholangiomas | ♀ 411, ♂ 534 | ♀ 534 | ♂ **285** |
| Hepatomas | — | ♀ **293** | — |
| Kidney : | | | |
| Hypernephromas | — | ♀ 610, ♀ 540 | — |
| Lung : | | | |
| Adenomas and carcinomas | ♀ 392, ♀ 589 | — | ♂ **285**, ♂ 622 |
| Mammary gland : | | | |
| Carcinoma | ♀ **189**, ♀ 386, ♀ 411, ♂ 534, ♀ 562, ♀ 589 | ♀ **289**, ♀ 596 | — |
| Pituitary : | | | |
| Chromophobe adenoma | ♂ 534 | — | — |
| Stomach : | | | |
| Carcinomas | — | ♀ 540 | ♂ 622 |
| Intestine : | | | |
| Squamous-celled carcinoma | ♀ 562 | — | — |
| Uterus : | | | |
| Fibroma and carcinoma | ♀ 589 | ♀ 534 | — |
| Thymus : | | | |
| Lymphangioma | ♀ 562 | — | — |
| Ear : | | | |
| Basal-cell carcinoma | — | ♀ **293** | — |
| Neck : | | | |
| Carcinoma | ♀ **290** | — | — |
| Groin : | | | |
| Carcionma | ♀ 392 | — | — |
| Sub-maxillary gland : | | | |
| Carcinoma | ♂ 534 | — | — |
| Total number of rats examined post mortem | 12 | 7 | 12 |
| Total number of tumours | 17 | 9 | 4 |

The numerals denote the number of days between the first treatment and diagnosis of tumours post mortem.

A marked difference in behaviour between this compound and 4-dimethylaminostilbene (I) is revealed in that no cholangiomas appeared within 6 months of commencement of treatment in the animals on Diet C as was the case with the latter compound. With Compound (II), apart from the fact that again more tumours were recorded in the rats on Diet A, which is almost certainly related to the longer survival time of the animals, there appears to be no definite effect of diet on the nature or distribution of the tumours. A number of tumours appeared in less than 12 months, but these were of random distribution and were not confined to any particular type or to animals on a particular diet. The largest number of tumours of one type were mammary carcinomas in females (although one occurred in a male). Since no females were used with compound (I) it is not possible to compare the behaviour of the two stilbenes in this respect.

A summary of the type and sites of occurrence of multiple tumours induced by (II) is given in Table IV.

TABLE IV.—*Sites of Occurrence of Multiple Tumours in Rats Treated with 2′-Chloro-4-Dimethylaminostilbene.*

| Sex of rat. | Diet. | Number of days between first treatment and diagnosis of tumours post mortem. | Location and type of tumours. | | |
|---|---|---|---|---|---|
| Female | B | 293 | Liver (hepatoma). | | Ear (basal-cell carcinoma). |
| ,, | B | 540 | Kidney (carcinoma). | | Stomach (squamous-celled carcinoma). |
| ,, | A | 589 | Lung (carcinoma). | Mammary gland (2 carcinomas). | Uterus (carcinoma). |
| ,, | B | 534 | Liver (cholangioma). | | Uterus (fibroma). |
| ,, | A | 562 | Intestine (carcinoma). | | Mammary gland (2 carcinomas). |
| ,, | A | 411 | Liver (cholangioma). | | Mammary gland (2 carcinomas). |
| ,, | A | 392 | Lung (bronchogenic carcinoma). | | Groin (carcinoma). |
| Male | A | 534 | Liver (cholangioma). | Pituitary (chromophobe adenoma). | Submaxillary gland (carcinoma). |

DISCUSSION.

A definite effect of diet on the carcinogenic action of the aminostilbene derivatives investigated is seen in the very short latent period required for the induction of cholangiomas in rats maintained on the diet containing 5 per cent protein with addition of methionine (Diet C). In the group of animals treated with 4-dimethylaminostilbene (I) whilst maintained on this diet, a total of 7 cholagiomas was found, 4 of which appeared within 3 months and 6 before the lapse of 11 months. No other tumours of any type were observed in less than a year from commencement of treatment with this compound in animals maintained on any of the diets.

The low protein Diet C therefore favours the development of cholangiomas, but not of other tumours in rats treated with 4-dimethylaminostilbene (I).

No such effect was observed in the animals treated with 2′-chloro-4-dimethylaminostilbene (II). Only one cholangioma was found in the rats maintained on Diet C, and although this was detected after about 9½ months it was not the earliest tumour to be induced by this compound. A mammary carcinoma was observed after about 6½ months' treatment in the group of rats maintained on the 20 per cent protein Diet A. A carcinoma of the neck in the Diet A animals, a mammary carcinoma, hepatoma and a basal-cell carcinoma of the ear in Diet B

animals, also a lung adenoma and a cholangioma in Diet C animals were all found within about 10 months after commencement of treatment with (II). No preferential effect on any of these diets in reducing the latent period of tumour development in rats treated with 2′-chloro-4-dimethylaminostilbene can therefore be claimed. The behaviour of 4-dimethylaminostilbene in respect of its liver tumour-inducing action is very similar to that of 4-dimethylaminoazobenzene, which it resembles closely in structure. Induction of liver tumours by both compounds is favoured by the same low protein diet, and in both cases it is the latent period required for induction of cholangiomas rather than of hepatomas which is shortened. The diet effect is so marked with 4-dimethylaminoazobenzene that in the experiment referred to previously which is now being carried out by Miss S. Charter, all the animals maintained on Diet C have developed cholangiomas in less than 5 months, whereas no tumours have as yet been detected (8 months) in rats receiving the 20 per cent protein Diet A.

With regard to the general carcinogenic action of the two aminostilbene derivatives (I) and (II), a variety of tumours have been found in 15 different organs. These include most of the types of tumours observed by Haddow, Harris, Kon and Roe (1948), and a number of tumours not previously described. A large number of sarcomas were found by the above authors, who administered the aminostilbenes by subcutaneous injection. No tumour of this type has been observed in the present experiment where the intraperitoneal route of administration was used.

In the paper by Haddow, Harris, Kon and Roe (1948) attention was drawn to the causation by a number of aminostilbene derivatives of haemolymph changes similar to those described by Latnitzki and Woodhouse (1942, 1944) in rats treated with carcinogenic hydrocarbons. The latter authors suggested that the ability of the hydrocarbons to produce such changes might in some way be connected with their carcinogenic action. The similar behaviour of the carcinogenic aminostilbenes has tended to support this suggestion, and Haddow *et al.* (1948) state that although the real significance of the phenomenon of the formation of haemolymph nodes is not at all apparent, it provided an additional reason for investigating the carcinogenic potency of such compounds.

Recent work by the present author may tend to throw considerable light on the significance of these so-called haemolymph changes in relation to carcinogenesis. This work is still in progress and will be fully described later, but it is felt that a brief reference to it in relation to the aminostilbenes should be made here. In studying the toxicity of a series of carcinogenic $\alpha\ \omega$ dimethane sulphonoxyalkanes, it was found that the treated rats develop a general haemorrhagic state and the immediate cause of death is usually a massive haemorrhage in the stomach. With sub-lethal doses it was noted by Miss C. Barrett that the haemorrhagic state could be easily demonstrated by plucking the fur from a small area on the chest of the animal. If positive a number of petechial haemorrhages appear almost immediately. The appearance of stomach haemorrhages and the fact that a marked haemolymph change always accompanies the haemorrhagic state suggested that the haemolymph changes observed by Haddow *et al.* (1948) with aminostilbenes might also be part of a general haemorrhagic state, particularly since some of these compounds also caused haemorrhage in the pyloric region of the stomach. This does appear to be the case, since with a dose of 85 mg./kg. i.p. of 4-dimethylaminostilbene a strongly positive " plucking test " was observed after 8 to 10 days. At post-mortem examination marked haemolymph changes were observed, and numerous small haemorrhages were evident in many organs, including lungs, testes, seminal vesicles, stomach, intestines, etc.

The haemorrhagic state induced by these compounds is obviously related to their effects on the blood, particularly—as is well known in the case of X radiation—to the diminution in the number of platelets. The haemolymph changes shown by carcinogens if, as appears to be the case with aminostilbene derivatives, they are related in some degree to a more general haemorrhagic state, may represent one aspect of the general growth inhibiting properties of these agents manifested here by prevention of cell division in the normal processes of haemopoiesis.

The author is greatly indebted to Dr. E. Horning who has carried out the pathological and histological examinations.

This investigation has been supported by grants to the Royal Cancer Hospital and Chester Beatty Research Institute from the British Empire Cancer Campaign, the Jane Coffin Childs Memorial Fund for Medical Research, the Anna Fuller Fund and the National Cancer Institute of the National Institutes of Health, U.S. Public Health Service.

### REFERENCES.

Elson, L. A.—(1947) *Biochem. J.*, **41**, XXI.—(1948) *Acta Un. int. Cancr.*, **6**, 396.
*Idem* and Haddow, A.—(1947) *Brit. J. Cancer*, **1**, 97.
*Idem* and Warren, F. L.—(1947) *Ibid.*, **1**, 86.
Haddow, A., Harris, R. J. C., Kon, G. A. R., and Roe, E. M. F.—(1948) *Phil. Trans.*, **A**, **241**, 147.
Lasnitzki, A., and Woodhouse, D. L.—(1942) *Nature*, **150**, 660.—(1944) *J. Anat., Lond.*, **78**, 121.